# Exploring Life Instability’s Relationship to the Mental Health of Older Adults With HIV

**DOI:** 10.1093/geroni/igab046.1636

**Published:** 2021-12-17

**Authors:** Elliott Weinstein, Audrey Harkness, Gail Ironson, Cho-Hee Shrader, Dustin Duncan, Steven Safren

**Affiliations:** 1 Psychology, Miami, Florida, United States; 2 Department of Public Health Sciences, University of Miami, Florida, United States; 3 Department of Psychology, University of Miami, Florida, United States; 4 Mailman School of Public Health, Columbia University, New York, United States

## Abstract

The study is one of the first to examine both the prevalence of life instability among older adults with HIV (OAWH) in a community clinic and its relationship to their mental health. OAWH (N=623) from a community medical clinic completed an interviewer-administered assessment (English/Spanish) which included an additive Life Instability Index (LII) composed of indicators at the individual (e.g. education, housing instability, employment status) and community (e.g. poverty, transportation) levels. Participants were a mean age of 60 years (SD = 5.90) with the majority identifying as Black-non-Hispanic (65.9%), cisgender male (60.8%), and heterosexual (80.6%). Participants reported an average of 6.08 destabilizing factors (SD = 1.44). In multiple linear regression analyses LII was significantly related to increased substance use among participants (b= 0.08, p < 0.01), but not with anxiety or depression. An LII is an innovative approach to assess the relationship between OAWH’s mental health and social determinants of health.

